# Hepatocellular cancer selection systems and liver transplantation: from the tower of babel to an ideal comprehensive score

**DOI:** 10.1007/s13304-021-01078-4

**Published:** 2021-05-18

**Authors:** Jan Lerut, Maxime Foguenne, Quirino Lai

**Affiliations:** 1 Institute for Experimental and Clinical Research (IREC), Université Catholique de Louvain (UCL), Avenue Hippocrates 55, 1200 Brussels, Belgium; 2 University Hospitals Saint-Luc Université Catholique de Louvain (UCL), Brussels, Belgium; 3 General Surgery and Organ Transplantation Unit, Sapienza University of Rome, Rome, Italy

**Keywords:** Liver transplantation, Hepatocellular cancer, Score, Selection criteria, Recurrent tumor

## Abstract

The Milan criteria (MC) remain the cornerstone for the selection of patients with hepatocellular cancer (HCC) to be listed for liver transplantation (LT). Recently, several expanded criteria have been proposed to increase the transplantability of HCC patients without compromising their (oncologic) outcome. This paper aims to systematically review the different reported HCC-LT selection systems looking thereby at their ability to increase the number of transplantable patients and the overall survival and oncological outcome. A systematic review of the literature covering the period 1993 (date of the first reported HCC-LT selection system)–2021 identified 59 different inclusion criteria of HCC for LT. Among the 59 studies reporting HCC-LT selection systems, 15 (28.3%) were exclusively based on morphological aspects of the tumor; 29 (54.7%) included biologic, seven (13.2%) radiological, and two (3.8%) only included pathological tumor features. Overall, 31% more patients could be transplanted when adhering to the new HCC-LT selection systems. Despite the increased number of LT, 5-year patient and disease-free survival rates were similar between MC-IN and MC-OUT/new HCC-LT-IN criteria. A careful extension of the inclusion criteria should allow many more patients to access a potentially curative LT without compromising their outcome. The development of a widely accepted “comprehensive” HCC-LT Score able to offer a fair chance of justified transplantation to more patients should become a priority within the liver transplant community. Further studies are needed to develop internationally accepted, expanded selection criteria for liver transplantation of HCC patients.

## Introduction

Thomas Starzl designed liver transplantation (LT) to treat unresectable primary and secondary hepatobiliary tumors [1, 2]. The first 'successful' LT was performed on July 23, 1967, in a child presenting with a large hepatocellular cancer (HCC) in the context of biliary atresia. The child died after 400 days, during which time she underwent many reinterventions to treat both thoracic and abdominal tumor recurrences. Due to the lack of selection criteria, the concept of LT as the primary treatment of hepatobiliary malignancies was rapidly challenged because of the prohibitively high incidence of tumor recurrence [2, 3]. The 'oncological pendulum' reversed in the nineties. The indication for LT moved from large multifocal lesions to a more limited tumor burden. A tumor load restricted to ≤ three tumors having a diameter ≤ 3 cm (Paris criteria) or one tumor ≤ 5 cm (Milan criteria, MC) resulted in 5-year disease-free survival (DFS) rates of 70–80% [4, 5]. The MC became the international gold standard to select HCC patients for LT [6–8]. However, after some years of stabilized practice, it became clear that the MC were too strict, denying access for many patients to potentially curative therapy. Many Western teams worked at a cautious extension of the inclusion criteria. Conversely, many Eastern ones adopted a much more aggressive attitude fostered by the explosive development of living-donor-liver transplantation (LDLT) [9]. The search for 'the ideal' score was launched to give as many patients as possible access to a potentially curative oncological procedure without compromising outcomes. However, the co-existence of multiple scoring systems explains the heterogeneous treatment of HCC, leading to difficulties when interpreting short- and long-term outcomes, and access to LT varies widely among countries, continents, and allocation organizations.

This paper aims to systematically review the different HCC-LT selection systems developed, with the intent to investigate their impact in terms of access to LT without compromising overall survival and oncological results. Using the available data, a meta-analysis was also done to investigate the post-transplant recurrence rates reported using the MC vs. the expanded selection criteria.

## Materials and methods

### Search sources and study design

A systematic review of the published literature on the different HCC-LT selection systems developed was undertaken. The search strategy was performed following the preferred reporting items for systemic reviews and meta-analysis (PRISMA) guidelines [10].

The specific research question formulated in the present study included the following PICO components:

Patient: patient with a confirmed HCC undergoing a LT;

Intervention: LT adopting an expanded HCC-LT selection system;

Comparison: LT adopting a standard selection approach (typically, the MC);

Outcome: patient death and/or tumor recurrence.

A search of the PubMed and Cochrane Central Register of Controlled Trials Databases was conducted using the following terms: ("liver transplant*"[Title/Abstract] OR "living donor liver transplant*"[Title/Abstract]) OR “living donor” AND ("criteria"[Title/Abstract] OR "score"[Title/Abstract] OR "model"[Title/Abstract]) AND ("HCC"[Title/Abstract] OR "hepatocellular carcinoma"[Title/Abstract] OR "hepatocellular cancer"[Title/Abstract]) AND ("1993/01/01"[PDAT]: "2021/03/14"[PDAT]).

The search period was from "1993/01/01" to "2021/03/14". The systematic review considered only English studies that included human patients. The start of the search period corresponded to the first publication of an HCC-LT selection system by the Bismuth group [4].

Published reports were excluded based on several criteria: (a) data on animal models; (b) lacked enough clinical details; (c) had non-primary source data (e.g., review articles, non-clinical studies, letters to the editor, expert opinions, and conference summaries). In studies originating from the same center, possible overlapping of clinical cases was examined, and the most informative study was considered eligible for inclusion.

### Data extraction and definitions

Following a full-text review of the eligible studies, two independent authors (MF and JL) performed the data extraction and crosschecked all outcomes. When selecting articles and data extraction, potential discrepancies were resolved following a consensus with a third reviewer (QL). Collected data included: first author of the publication, reference number, center, year of publication, type of selection system (based on morphological, biological, radiological, or pathological aspects), number of cases, number of patients within the new selection system, number of cases within MC, number of patients exceeding MC, additive number and increased percentage of LT cases compared with the MC, 5-year overall and disease-free survival rates in new criteria-IN, MC-OUT/new criteria-IN, and new criteria-OUT cases and finally percentage of living donor LT.

As already reported, we stratified the selection systems identified in four groups according to the characteristics of the variables composing the scores. In detail: (a) “morphological” systems were based only on the radiology-derived tumor variables (i.e., number and dimensions); (b) “biological” systems also included biological markers derived from the blood tests; (c) “radiological” systems also included variables derived from the post-locoregional therapy response or the radiology-related tumor activity (i.e., PET avidity); and, (d) “histological” scores also included parameters connected with pre-LT biopsies.

### Quality assessment

Selected studies were systematically reviewed with the intent to identify potential sources of bias. The papers' quality was assessed using the Risk of Bias In Non-randomized Studies of Interventions (ROBINS-I) tool [11].

### Statistical analysis

The meta-analysis was performed using OpenMetaAnalyst. The statistical heterogeneity was evaluated with the Higgins statistic squared (*I*^2^). *I*^2^ value was considered indicative of heterogeneity: low = 0–25%; 26–50% = moderate; ≥ 51% = high. In the case of low-to-moderate (0–50%) heterogeneity, a fixed-effects model was used. The random-effects model was used when high heterogeneity was reported. The odds ratio (OR) and 95% confidence intervals (95% CI) were reported. A *P* value < 0.05 was considered indicative of statistical significance.

## Results

### Search results and study characteristics

**Fig. 1:**
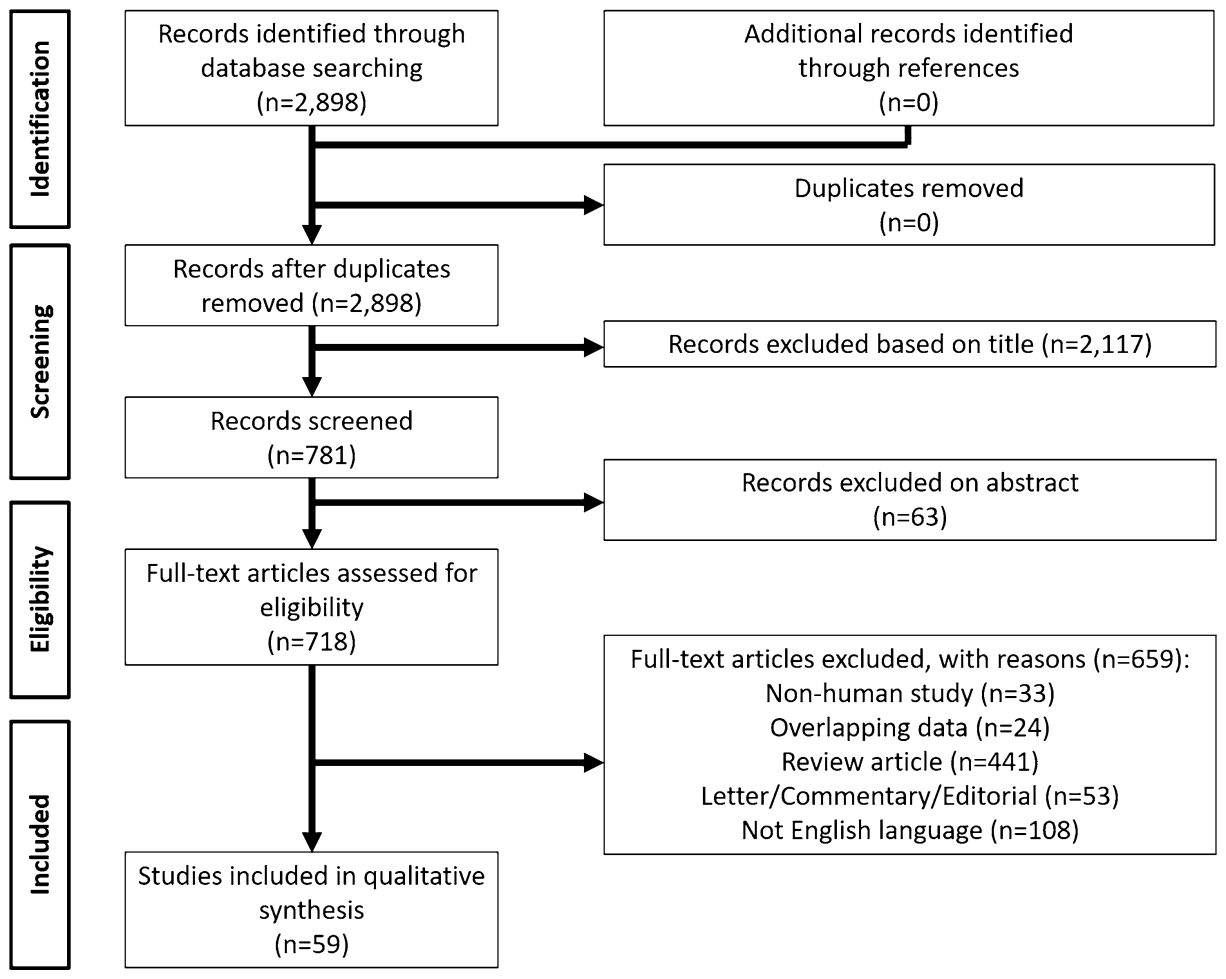
PRISMA flow diagram showing the article selection process

The PRISMA flow diagram schematically depicts the article selection process (Fig. 1). Among the 2898 articles screened, 59 studies reporting HCC-LT selection systems were identified [4, 5, 7, 8, 12–66].

The variables adopted for constructing the selection systems and selecting HCC patients for LT were as follows: 15 (25.4%) were exclusively based on morphological tumor characteristics; 34 (57.6%) on biological characteristics either alone or in combination with morphological features, eight (13.6%) on radiological features, and two (3.4%), on pathological characteristics only. More detailed information about the different variables used to construct a new selection system is displayed in Table 1 [4, 5, 7, 12–66].

**Table 1:** HCC and LT Scores based on the different combinations of tumor morphology, biology, radiology, and pathology

Ref	Author		Center	Year	Morphology		Biology	Radiology	Pathology
Morphologic only HCC characteristics									
[4]	Bismuth		Paul Brousse	1993	≤ 2 T with largest T diam ≤ 3 cm		–	–	–
[5]	Mazzaferro		Milan	1996	1 T ≤ 5 cm OR 2–3 T ≤ 3 cm each		–	–	–
[12]	Iwatsuki		Pittsburgh	2000	No bilobarity, largest T diam ≤ 5 cm, no vascular invasion		–	–	–
[7]	Yao		UCSF	2001	1 T ≤ 6.5 cm OR 2–3 T ≤ 4.5 cm each with TTD ≤ 8 cm		–	–	–
[13]	Kneteman		Edmonton	2004	1 T ≤ 7.5 cm OR multiple T each ≤ 5 cm		–	–	–
[14]	Jonas		Berlin	2007	largest T diam ≤ 6 cm with TTD ≤ 15 cm		–	–	–
[15]	Onaca		Dallas	2007	1 T ≤ 6 cm OR 2–4 T ≤ 5 cm each		–	–	–
[16]	Sugawara		Tokyo	2007	≤ 5 T with each T ≤ 5 cm		–	–	–
[17]	Herrero		CUN Navarra	2008	1 T ≤ 6.5 cm OR 2–3 T ≤ 5 cm each		–	–	–
[18]	Lee		ASAN Seoul	2008	≤ 6 T with largest T diam ≤ 5 cm		–	–	–
[19]	Silva		Valencia	2008	1–3 T ≤ 5 cm each with TTD ≤ 10 cm		–	–	–
[20]	Fan		Shanghai Fudan	2009	1 T ≤ 9 cm OR 1–3 T ≤ 5 cm each with TTD ≤ 9 cm		–	–	–
[21]	Li		Sichuan	2009	TTD ≤ 9 cm		–	–	–
[8]	Mazzaferro		Up-to-7	2009	Number T + largest T diam ≤ 7		–	–	–
[22]	Choi		CUK Seoul	2012	≤ 7 T with largest T diam ≤ 7 cm		–	–	–
Combined morphologic and biological HCC characteristics									
[23]	Ito		Kyoto	2007	≤ 10 T with each T ≤ 5 cm		DCP ≤ 400 mAU/mL	–	–
[24]	Todo		Hokkaido	2007	Milan criteria		AFP ≤ 200 ng/mL AND DCP ≤ 100 mAU/mL	–	–
[25]	Kwon		SMC Seoul	2007	Largest T diam ≤ 5 cm, no number restriction		AFP ≤ 500 ng/mL	–	–
[26]	Yang		Seoul	2007	≤ 3; 3.1–5; 5.1–6.5; > 6.5 cm / 1; 2–3; 4–5; 6 T		AFP < 20; 20–200; 200–1000; > 1000 ng/mL	–	–
[27]	Xu		Hangzhou	2016	TTD ≤ 8 cm		If TTD > 8 cm: AFP ≤ 400 ng/mL + grade I/II	–	–
[28]	Taketomi		Kyushu	2009	largest T diam ≤ 5 cm		DCP ≤ 300 mAU/mL	–	–
[29]	Vibert		Villejuif—Paul Brousse	2010	No restrictions		AFP slope < 15 ng/ml/month	–	–
[30]	Duvoux		Créteil	2012	AFP-Model, low risk ≤ 2		AFP model low risk ≤ 2	–	–
[31]	Lai		Rome	2012	TTD ≤ 8 cm		AFP ≤ 400 ng/mL	–	–
[32]	Choi		CUK Seoul	2013	Largest T diam ≤ 5 cm		AFP ≤ 100 ng/mL	–	–
[33]	Li		Sichuan	2013	TTV < 172 cm3		If TTV > 172 cm3: lymphocytes ≤ 30%	–	–
[34]	Yoshizumi		Fukuoka	2013	Number T + largest T diam ≤ 8		NLR ≤ 4	–	–
[35]	Na		CUK Seoul	2014	No restrictions		CRP ≤ 1 AND NLR ≤ 6	–	–
[36]	Wan		Shanghai	2014	No restrictions		CA 19.9 ≤ 100 ng/mL AND AFP ≤ 400 ng/mL	–	–
[37]	Wan		Shanghai	2014	Largest T diam ≤ 10 cm		AFP ≤ 400 ng/mL	–	–
[38]	Shindoh		Tokyo bis	2014	Tokyo criteria		AFP ≤ 250 ng/mL AND DCP ≤ 450 mAU/mL	–	–
[39]	Kashkoush		Alberta	2014	TTV ≤ 115 cm3		AFP ≤ 400 ng/mL	–	–
[40]	Kim		SMC criteria	2014	≤ 7 T with each T ≤ 6 cm		AFP ≤ 1000 ng/mL	–	–
[41]	Xiao		Chengdu	2015	Hangzhou criteria		NLR ≤ 4	–	–
[42]	Yang		Pusan University	2016	No restrictions		AFP < 200 ng/mL AND DCP < 200 mAU/mL	–	–
[43]	Lee JH		MoRAL South Korea	2016	No restrictions		11*square root(DCP) + 2*(square root(AFP); low MoRAL < 314.8	–	–
[44]	Kim SH		ASAN Seoul AMC group	2016	No restrictions		AFP < 150 ng/mL AND DCP < 100 mAU/mL	–	–
[45]	Xia		Zheijiang	2017	Hangzhou criteria		PLR ≤ 120	–	–
[46]	Grat		Warsawa	2017	Up to 7/UCSF		AFP ≤ 100 ng/mL	–	–
[47]	Halazun		MoRAL New York	2017	Pre-MoRAL: NLR > 5 = 6 points; Largest T diam > 3 cm = 3 points Post-MoRAL: Grade 4 = 6 points; Vascular invasion = 2 points; Largest T diam > 3 cm = 3 points; T > 3 = 2 points		AFP > 200 ng/mL = 4 points	–	–
[48]	Halazun		NYCA New York-UCLA	2018	Largest T diam < 3 cm = 0 points; 3–6 cm = 2 points, > 6 cm = 4 points / 1 T = 0 points; 2–3 T = 2 points; ≥ 4 T = 4 points		AFP < 200 (always) = 0 points; AFP-responder = 2 points; AFP non-responder = 3 to 6 points	–	–
[49]	Mazzaferro		Metroticket 2.0 Italy (Training)/Fudan Shanghai (Validation)	2018	Up to 7; Up to 5; Up to 4		AFP < 200; 200–400; 400–1000	–	–
								–	–
[50]	Shimamura		5–5-500	2018	≤ 5 T with each T ≤ 5 cm		AFP ≤ 500 ng/mL	–	–
[51]	Fiel		HALT Cleveland	2019	(2.31*ln(AFP)) + (1.33*(TBS)) + (0.25*MELDNa) − (5.57*Asia)				
[52]	Ince		Malatya	2020	MC-in within the criteria. If MC-out: Largest T diam ≤ 6 cm		MC-in within the criteria. If MC-out: AFP ≤ 200 ng/mL + GGT ≤ 104 IU/L + grade I/II	–	–
[53]	Daoud		UNOS data	2021	Milan criteria and AFP ≤ 2500 ng/mL UCSF criteria ≤ 150 ng/mL			–	–
[54]	Mazzotta		AFP-Model modified	2021	High-risk for number of nodules: > 5 instead of > 3			–	–
[55]	Goldberg		LiTES-HCC	2021	Age, bilirubin, chronic kidney disease, INR, diabetes, etiology of liver disease, difference TTD at LT vs. waiting list, difference AFP at LT vs. waiting list, pre-LT location, pre-LT ventilation			–	–
[56]	Hwang		ADV < 5log	2021	Log10(AFP* DCP*total volume)			–	–
Combined morphologic, biological, and radiological HCC characteristics									
[57]		Roayaie	Mount Sinai New York	2002	1 T > 5 cm	–		TACE	–
[58]		Kornberg	Munich	2012	–	–		PET-CT negative	–
[59]		Lai	EurHeCaLT	2013	Milan criteria	AFP slope ≥ 15 ng/mL/month		mRECIST progression	–
[60]		Kornberg	Munich	2014	–	–		Bridging response necrosis > 50%	–
[61]		Lee	NCCK	2016	TTD ≤ 10 cm	–		PET-CT SUV < 3.08	–
[62]		Hsu	Koahsiung Chang Gung—Taiwan	2016	UCSF criteria	–		PET-CT negative (TNR < 2)	–
[63]		Lai	TRAIN Brussels (Training)/Ancona (Validation)	2016	0.988 if mRECIST-PD + 0.838 if AFP slope > 15 ng/mL/month + 0.452 if NLR > 5.0 + 0.03*WT (in months) Low TRAIN < 1.0				–
[64]		Bhangui	Medanta	2021	UCSF criteria/Milan criteria	AFP ≥ 100 ng/mL		PET-CT [18F]FDG avidity	–
Only pathological HCC characteristics									
[65]		Cillo	Padua	2004	No tumor size/tumor number restriction		–	–	Moderately or well differentiated tumor
[66]		DuBay	Toronto	2011	No tumor size/tumor number restriction		–	–	No systemic symptoms. Not poorly differentiated if MC-OUT

*Ref* reference, *HCC* hepatocellular cancer, *T* tumor, *TTD* total tumor diameter, *DCP* des-gamma-carboxy prothrombin, *AFP* alpha-fetoprotein, *B* biology-related parameters, *TTD* total tumor diameter, *TTV* total tumor volume, *TBS* tumor burden score, *TACE* trans-arterial chemo-embolization, *PET* positron emission tomography, *CT* computed tomography, *AFP* alpha-fetoprotein, *mRECIST* modified response evaluation criteria in solid tumors, *RF* risk factors, *TTD* total tumor diameter, *SUV* standardized uptake value

As for the period of publication, only two studies (3.4%) were published before 2000, [4, 5] 21 (35.6%) during the decade 2000–2009, and 36 (61.0%) during the decade 2010–2021. Interestingly, all but one study based only on morphological tumor characteristics was published before 2010 [23]. The geographical distribution of the articles was as follows: Asia 30 (50.8%), Europe 17 (28.8%), and North America 12 (20.4%). In 22 (37.3%) papers, HCC-LT selection systems were developed in the field of LDLT. In 47 (79.7%) studies, the MC status was reported, thereby comparing the respective proposed new selection systems. According to the data reported, the MC status was estimable in only one (1.7%) report.

### Qualitative assessment of the included studies

**Fig. 2:**
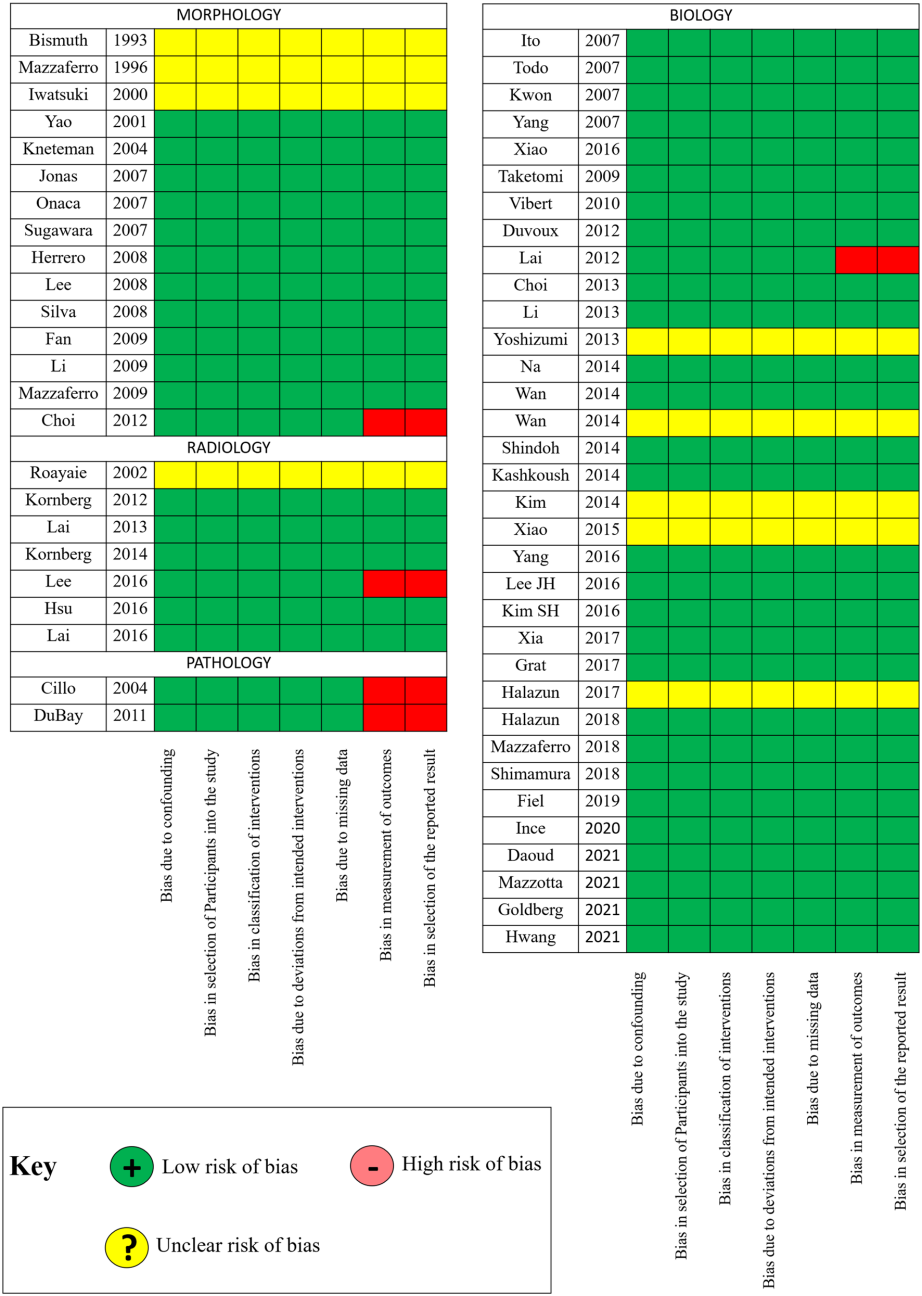
ROBINS-I qualitative assessment of the included studies

Results from the qualitative assessment of the included studies are shown in Fig. 2. Overall, 9 (15.3%) studies presented an unclear risk of bias due to the absence of data from a comparative group; in 5 (8.5%) studies, data comparing the outcome of the proposed new selection system with a comparative one were incompletely reported, leading to a potentially high risk of bias.

### Review of the eligible studies: the 'tower of Babel' of the selection systems

Data concerning the results observed in the analyzed selection systems are displayed in Table 2 [4, 5, 7, 8, 12–66].

**Table 2:** HCC and LT: overall and disease-free survival rates—results of the different scores

Ref	Center	Nr	New IN	MC-IN	MC-OUT	Additive LT cases				5-yr OS %				5-yr DFS %			LDLT (%)
						Nr		%		New IN	MC-OUT New IN	New OUT	New IN		MC-OUT/new IN	New OUT	
Only morphologic hcc characteristics																	
[4]	Paul Brousse	60	28	28	0	0		0		83 (3 yr)	–	–	83 (3 yr)		–	–	–
[5]	Milan	48	35	35	13	0		0		85 (4 yr)	–	50 (4 yr)	92 (4 yr)		–	59 (4 yr)	–
[12]	Pittsburgh	318	NA	NA	NA	NA		NA		NA	NA	NA	100, 61, 40, 5, 0 in the five classes				–
[7]	UCSF	70	60	46	24	14		20		75	–	50 (1 yr)	–		–	–	–
[13]	Edmonton	40	40	19	21	21		53		83 (4 yr)	–	–	77 (4 yr)		–	–	–
[14]	Berlin	21	21	8	13	13		62		68 (3 yr)	–	–	64 (3 yr)		–	–	21 (100)
[15]	Dallas	1038	769	631	407	138		18		–	–	43	64		–	–	–
[16]	Tokyo	78	72	68	10	4		6		75	–	–	94 (3 yr)		–	50 (3 yr)	78 (100)
[17]	CUN Navarra	71	71	47	24	24		34		74	–	–	–		–	–	–
[18]	ASAN Seoul	221	186	164	57	22		12		76	–	19	85		91 (3 yr)	26 (3 yr)	221 (100)
[19]	Valencia	257	211	231	26	-20		-9		67	–	40	89		–	57	–
[20]	Shanghai Fudan	969	570	394	575	176		31		78	76	–	53		46	–	–
[21]	Sichuan	165	49	24	140	25		51		83	–	–	69		–	–	–
[8]	Up-to-7	1525	727	444	1112	283		39		71	71	48	–		–	–	121 (8)
]22]	CUK Seoul	199	172	128	71	44		26		72	–	30	87		–	38	199 (100)
Tot^a^	–	4762	3011	2267	2493	744		33		–	–	–	–		–	–	–
Combined morphologic and biological HCC characteristics																	
[23]	Kyoto	125	78	70	55	8	10		87		–	34	95		93	40	125 (100)
[24]	Hokaido	551	351	343	208	8	2		–		–	–	96		79	40	551 (100)
[25]	SMC Seoul	139	114	99	40	15	13		87		–	23	88		–	42	139 (100)
[26]	Seoul	63	49	40	23	9	23		84 (3 yr)		–	0 (3 yr)	84 (3 yr)		–	25 (3 yr)	63 (100)
[27]	Hangzhou	6012	3798	2626	3386	1172	45		62		62	33	57		57	28	–
[28]	Kyushu	90	85	36	54	49	58		83		–	20	87		–	0	90 (100)
[29]	Villejuif—Paul Brousse	153	127	99	54	28	22		77		–	54	74		58	47	–
[30]	Créteil	391	320	296	95	24	8		68		–	48	91		85	49	–
[31]	Rome	158	143	117	41	26	18		NA		–	–	74		–	52	–
[32]	CUK Seoul	224	140	133	91	7	5		82		–	66	89		–	66	224 (100)
[33]	Sichuan	216	164	93	123	71	43		NA		–	–	76		76	48	60 (28)
[34]	Fukuoka	104	58	52	52	6	10		NA		–	–	100		-	15 (3 yr)	104 (100)
[35]	CUK Seoul	224	204	133	91	71	35		83		76	–	91		81	-	224 (100)
[36]	Shanghai	226	137	107	119	30	22		75		79	24	79		75	29	–
[37]	Shanghai	130	35	0	130	35	–		74		–	–	74		–	–	–
[38]	Tokyo bis	124	110	80	44	30	27		88		–	20	98		–	20	124 (100)
[39]	Alberta	115	88	61	54	27	31		82		82	–	88		–	55	–
[40]	SMC criteria	180	146	NA	NA	NA	NA		–		–	–	90		–	57	157 (87)
[41]	Chengdu	305	27	NA	NA	NA	NA		62		–	12	75		–	10	–
[42]	Pusan University	88	65	59	23	6	9		89 (3 yr)		–	80 (3 yr)	90 (3 yr)		88 (3 yr)	43 (3 yr)	72 (82)
[43]	MoRAL South Korea	566	NA	361	205	NA	NA		83		83	–	68		66	–	
[44]	ASAN Seoul AMC group	461	397	305	156	92	23		83		–	63	92		–	55	461 (100)
[45]	Zheijiang	348	184	144	204	40	22		–		–	–	73		73	15	41 (12)
[46]	Warsawa	240	172	143	97	29	17		75		82	55	92		100	45	–
[47]	MoRAL New York	339	NA	226	113	NA	NA		–		–	–	Pre: gr 1 = 99; gr 2 = 70 Post: gr 1 = 97; gr 2 = 75		78	–	–
[48]	NYCA New York-UCLA	1450	1416	1215	235	201	14		75 low risk		–	40	90 low risk 72 high risk		–	72	–
[49]	Metroticket 2.0 Italy (Training)	1018	NA	NA	NA	NA	NA		80		–	50	90		–	45	–
	Fudan Shanghai (Validation)	341	NA	NA	NA	NA	NA		81		–	60	86		93	60	–
[50]	5–5-500	965	735	664	301	71	10		76		–	52	73		–	43	965 (100)
[51]	HALT Cleveland	4089	NA	3059	1030	NA	NA		82 HALT < 5 32 HALT > 35		–	–	91 HALT < 5 30 HALT > 35		–	–	–
[52]	Malatya	215	104	152	63	41	19		80		72	37	–		–	–	NA
[53]	UNOS data	11,928	NA	11,555	373	NA	NA		MC + AFP ≤ 2500: 59 MC + AFP ≤ 2500: 55		NA	MC + AFP ≤ 2500: 37 MC + AFP ≤ 2500: 36					NA
[54]	AFP-Model modified	143	124	NA	NA	8	6		78		–	24	73		–	0	–
[55]	LiTES-HCC	6502	NA	NA	NA	NA	NA		86 score group 4 67 score group 1		NA	NA	NA		NA	NA	–
[56]	ADV < 5log	843	731	658	185	73	9		90		–	63	84		–	45	843 (100)
Tot^a^	-	13,655	9805	7725	5924	2169	16		–		–	–	–		–	–	–
Combined morphologic, biological, and radiological hcc characteristics																	
[57]	Mount Sinai New York	43	43	0	43	43	–		44		–	–	48		–	–	–
[58]	Munich	91	56	57	34	−1	−2		–		–	–	81		81	21	13 (14)
[59]	EurHeCaLT	422	398	306	116	92	23		88		84	55	90		87	42	–
[60]	Munich	93	59	57	36	2	3		MC-OUT Response 80		–	–	96		80	21	–
[61]	NCCK	280	164	132	148	32	20		85		-	60	84		-	44	280 (100)
[62]	Koahsiung Chang Gung—Taiwan	147	83	80	67	3	4		-		-	-	94		30	-	147 (100)
[63]	TRAIN Brussels (Training)	179	152	136	43	16	11		68 ITT		70 ITT	24 ITT	91		70	70	–
	Ancona (Validation)	110	97	70	40	27	28		67 ITT		70 ITT	21 ITT	86		73	0	–
[64]	Medanta	300	263	150	150	113	38		–		–	–	89		71	41	300 (100)
Tot	–	1665	1315	988	677	327	20		–		–	–	–		–	–	–
Pathological only HCC characteristics																	
[65]	Padua	48	48	33	15	15	31		75		–	–	92		–	–	–
[66]	Toronto	294	289	189	105	100	35		79		–	–	76		–	–	–
Tot	–	342	337	222	120	115	34		–		–	–	–		–	–	–

*Nr* number, *MC* Milan criteria, *LT* liver transplant, *OS* overall survival, *yr* years, *DFS* disease-free survival, *LDLT* living-donor-liver transplant, *HCC* hepatocellular cancer, *ITT* intention-to-treat

^a^Calculated using only the studies with all the available data

When considering the 48 (81.4%) studies in which sufficient information was available about the MC status, a total of 20,409 cases were reported, 14,453 of them met the new criteria, and 11,189 were MC-IN.

**Fig. 3:**
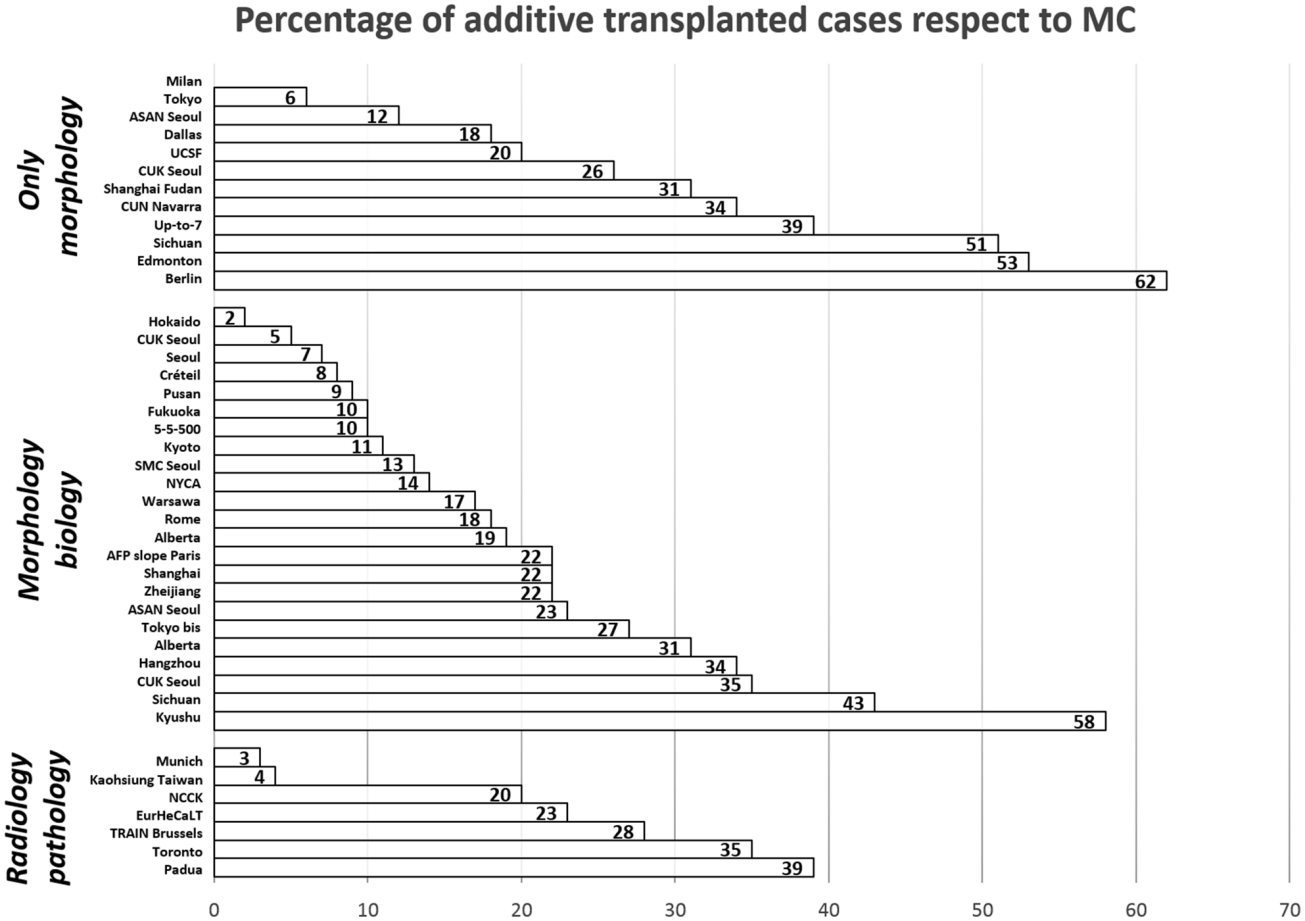
Percentage of supplementary liver transplantations compared to the Milan criteria when using new expanded criteria

Overall, a total number of 3353 new criteria-IN/MC-OUT cases were reported leading to a 16% increase of transplanted HCC patients. Apart from two reports [19, 58], all proposed expanded selection systems aimed to widen the inclusion criteria. This intent led to an increase in transplanted patients from 2 to 62% compared with the MC. (Table 2 and Fig. 3).

**Fig. 4:**
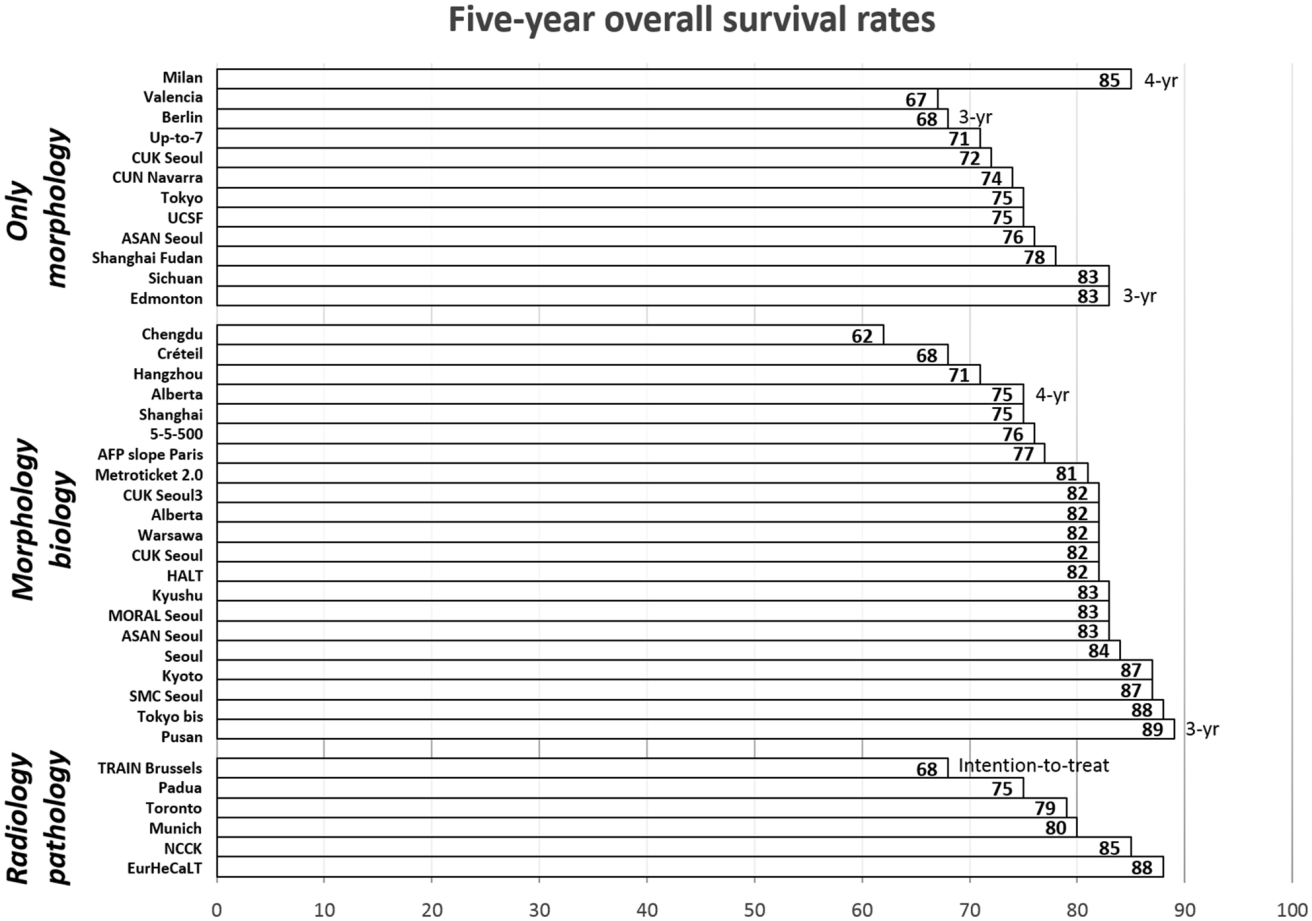
5-year overall survival rates in the different reported HCC criteria

**Fig. 5:**
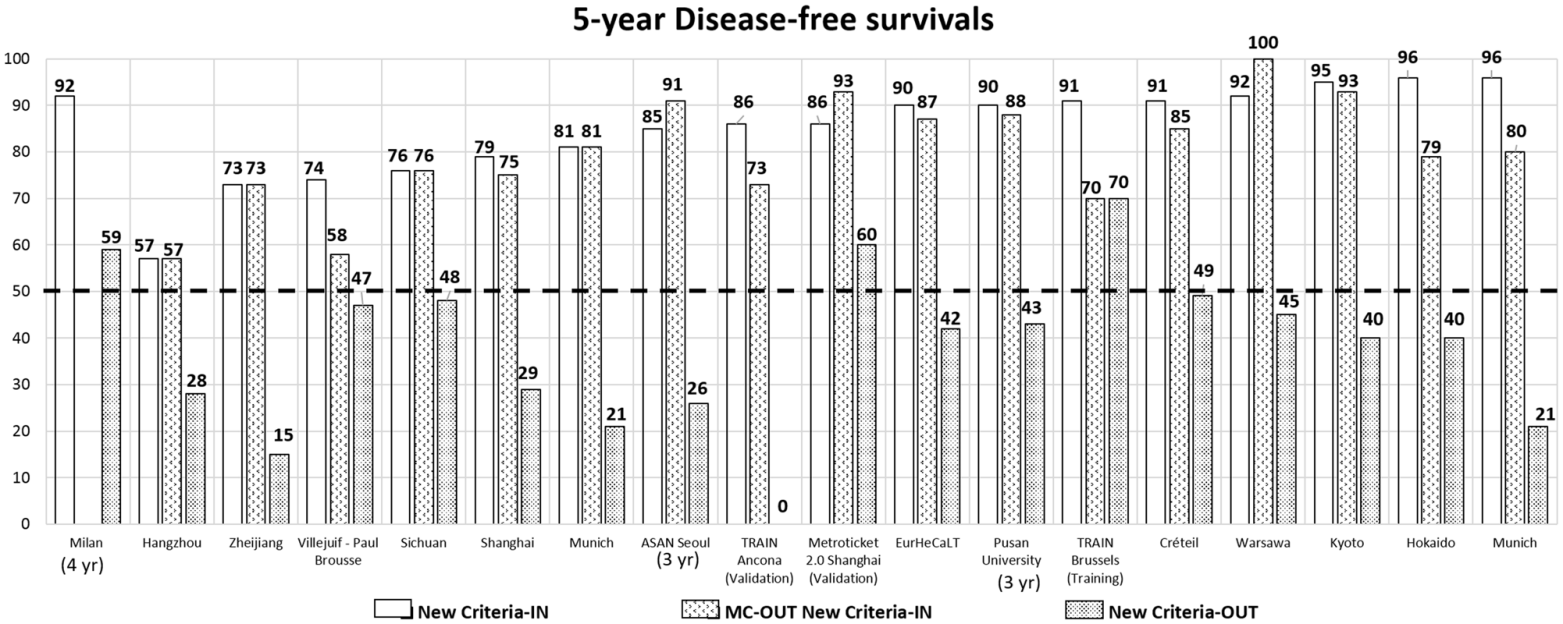
5-year disease-free survival rates in patients within the Milan criteria, without the Milan criteria but within the new expanded criteria or exceeding the new criteria

Despite the increased number of transplants, the results were only moderately compromised. Interestingly, if the tumor load was within the respective new criteria, 5-year patient survival rates were always superior to 50% (range: 62–90%) (Table 2 and Fig. 4). When adhering to the new criteria, excellent 5-year DFS rates were also obtained. Conversely, DFS dropped each time below 50% if the new selection system was overruled (Table 2 and Fig. 5).

### Meta-analysis for the post-transplant recurrence

**Fig. 6:**
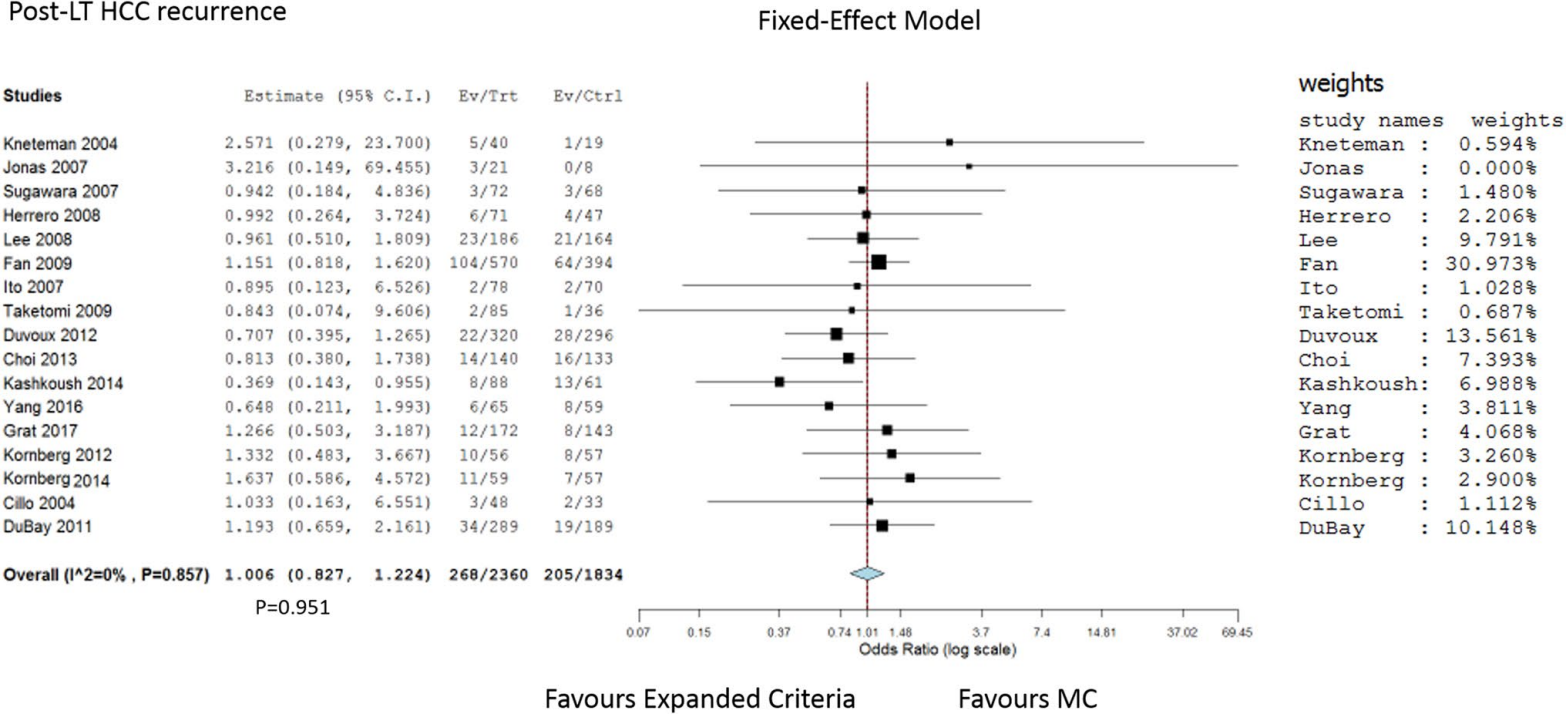
Forest plot and meta-analysis on the post-transplant recurrence: Milan criteria vs. enlarged selection criteria

Only seventeen papers reported the post-transplant recurrence data required to perform a meta-analysis to compare the MC vs. the expanded criteria [13, 14, 16–18, 20, 23, 28, 30, 32, 39, 42, 46, 58, 60, 65, 66]. When the papers were investigated, no heterogeneity was reported (*I*^2^ = 0, *P* = 0.857). A total of 1834 patients meeting the MC (205 recurrences, 11.2%) were compared with 2360 patients meeting the different proposed expanded selection systems (268 recurrences, 11.4%). No statistical significance was reported between the two groups (OR = 1.006, 95% CI = 0.827–1.224; *P* = 0.951), although a + 28.7% of transplantable cases was observed using the expanded criteria (Fig. 6).

## Discussion

The data observed in the present systematic review confirm that a careful extension of the inclusion criteria may allow many patients to access a potentially curative LT without seriously compromising the outcome.

The first HCC-LT selection system was ‘officially’ born in 1996 when Mazzaferro proposed the MC, achieving a 4-year DFS rate of 92% [5]. Despite the low number of patients reported (*n* = 48), the retrospective design of the study, and the absence of a control group, the MC still rule access of patients to transplant waiting lists more than 30 years later.

MC represent a very efficacious system for selecting HCC patients waiting for LT thanks to its super-selective ability. This is probably the main reason why the MC remain the most valuable benchmark considered in the setting of LT oncology, even in the presence of a large number of studies considering other more sophisticated parameters. However, the strength of the MC contemporaneously represents its weakness: in fact, the super-selection of the MC excludes a too high number of potentially transplantable patients from a curative strategy.

In 2001, the University of California San Francisco (UCSF) group was the first to challenge the MC. Similar survival rates were obtained using their new criteria, the critical difference being that 20% more patients were able to access a curative LT [7]. Up to now, 59 different HCC scoring systems have been proposed in the setting of HCC and LT [4, 5, 7, 8, 12–66].

All the criteria “extending” the MC can be grouped under the “Metroticket” definition again introduced by the Milan group: the further the trip (namely, the larger the tumor burden), the more expensive the ticket (namely, the higher the post-LT recurrence rate) [8].

Initially, the extension of inclusion criteria for LT was exclusively based on morphological criteria, namely tumor number and diameter [4, 5, 7, 8, 12–22]. In 2007, the Kyoto group [23] for the first time demonstrated that the morphology-alone selection approach was overruled by two fundamental principles of modern oncology, namely the necessity to a) combine tumor morphology and biology and b) evaluate the response to neo-adjuvant therapies to address tumor aggressiveness and behavior [23–66]. The Kyoto group showed that a successful LT could be achieved in patients harboring up to ten tumors on the condition that the tumor marker Protein Induced by Vitamin K Absence-II (PIVKA-II) was ˂400 mAU/mL [23].

Other Asian groups elaborated on this concept during the same period by introducing alpha-fetoprotein (AFP) levels in their selection systems [24–26]. Several Japanese and South-Korean centers raised AFP and PIVKA-II sensitivity by contemporaneously using these markers [24, 38, 42–44, 56]. Also centers from Western countries progressively introduced AFP to select HCC patients, with cut-off levels ranging from 100 to 2,500 ng/mL [30, 31, 39, 46–49, 51, 53–55]. Later, inflammatory markers such as neutrophil- (NLR) and platelet-to-lymphocyte (PLR) ratios were added for further refinement [33–35, 41, 45, 47, 63]. Recently, the radiological response has also been introduced as a useful parameter in selecting HCC cases. For example, the progressive disease after treatment using the mRECIST criteria has been adopted in several studies for predicting the risk of poor post-transplant clinical course [59, 61]. Also the tracer uptake by the HCC at PET-CT scanning has been added as a good prognostic factor in some selection systems [58, 61, 62, 64].

The use of radiological response as a selective tool is the direct consequence of the everyday use of locoregional therapies before transplant, both in the settings of bridging and downstaging [67]. Thanks to the direct effect of these treatments, the selection process has further moved from static to dynamic tumor evaluation. AFP slope ˂15 ng/ml/month [29, 59, 63] and any morphological response on imaging using the modified-Response evaluation criteria in solid tumors (mRECIST) criteria are favorable prognostic factors [59, 63].

It is interesting to note that almost all the proposed expanded HCC-LT selection systems permit the transplantation of more patients without seriously compromising their long-term outcome. This evidence is also confirmed in the meta-analysis performed, in which very similar recurrence rates were observed comparing the MC vs. the new criteria, despite a + 28.7% of transplantable cases was reported using these enlarged systems.

It is of particular interest to note that the DFS rates of patients exceeding the MC but meeting the new selection systems were similar to those obtained in MC. The selection process driven by the new criteria identified a sub-group of MC-OUT patients benefitting from LT. Conversely, if the new selection systems were overruled (new criteria-OUT patients), 5-year DFS was always inferior to 50%, a number corresponding to an oncologically futile transplant procedure. [68, 69].

It is difficult to identify the best selection system to use among the proposed ones. The experiences gathered during the last three decades in both deceased and living donor LT in both Western and Eastern centers indicate that the development of a universally acceptable selection system is within reach. The “ideal” HCC-LT score should incorporate scientifically reliable, pre-operatively available, easy-to-use, dynamic, morphological plus biological, tumor characteristics.

To further improve the selection process, four different matters need to be explored further. The first relates to the pre-transplant diagnosis of microvascular tumor invasion and poor tumor grading. Due to intra-tumor heterogeneity, tumor aggressiveness is challenging to capture with a biopsy [70]. PIVKA-II, a surrogate marker of vascular invasion, should be systematically implemented in clinical use in Western countries [71]. It is to be expected that radiomics will help to solve this shortcoming in the near future [72].

The second matter relates to the impact of LDLT in the treatment of HCC patients waiting for LT. LDLT not only represents a unique opportunity to increase the allograft pool (necessary to cope with the rising number of HCC patients), but most of all allow exploration of the effect of expanding the HCC inclusion criteria without harming non-tumor patients on the waiting list [73]. The role of LDLT in treating HCC patients will become increasingly important, because dropout risk is virtually eliminated [74]. Important in this (ethical) context is also the fact that recent technical developments have turned LDLT from a “high risk, high return” into a “low risk, high return” procedure [75]. These considerations imply that LDLT represents a fertile soil to explore further the role of transplantation in the cure of HCC patients. The time has come for the Western world to take up this challenge.

The third matter relates to integrating the concept of transplant benefit in HCC patient selection. Transplant survival benefit corresponds to the number of years gained by LT minus the number of years offered by alternative treatments from LT. Intention-to-treat transplant survival benefit adheres to the same concept, considering the gain in life expectancy, but from waiting list registration, thereby taking into consideration any possible therapy from the time of HCC diagnosis [76]. The identification of selection systems based on the concept of benefit should improve the selection process of HCC patients by identifying patients deserving LT and avoiding futile transplants in patients presenting with too advanced or too early tumor burdens.

Finally, any selection system should also consider the immunosuppression load of the HCC liver recipient. Immunosuppression cannot be disregarded in the context of LT for HCC, as it is the most relevant pro-oncogenic factor [77]. This consideration is especially critical when expanding the inclusion criteria, which, by definition, implies a larger tumor burden and a potentially higher risk of recurrence, and when dealing with remaining tumor tissue at the examination of the total hepatectomy specimen [78]. The development of more extensive inclusion criteria should be accompanied by strategies that aim to minimize the immunosuppressive load.

The present study has some limitations. As already underlined, some of the selected papers revealed an uncertain or high risk of bias. This limit is the consequence of the retrospective and non-randomized nature of all studies exploring the role of HCC-LT selection systems. Another limitation relates to the poor homogeneity of the different proposed selection systems, with only a minimal number of studies reporting their external validation. The significant absence of data available in the articles strongly limited our meta-analysis. Only 17/66 articles clearly stated the recurrence data required. Indeed, more homogeneous and more detailed studies are required for conducting such an investigation using more significant numbers.

## Conclusions

The development of a widely accepted “comprehensive” HCC-LT selection system is a necessity. To reach this goal, the development of new diagnostic technologies, more comprehensive implementation of living-donor-liver transplantation, and integration of the concept of benefit into the therapeutic scheme of HCC patients will be necessary. All these elements are essential to bring order to the chaos of selection systems and, more importantly, to offer the best possible treatment to the highest possible number of HCC liver patients. Hopefully, the tower of Babel of scores will disappear in the near future.

## Abbreviations

AFP: Alpha-fetoprotein
CI: Confidence intervals
DFS: Disease-free survival
HCC: Hepatocellular cancer
I2: Higgins statistic squared
LDLT: Living donor liver transplantation
LT: Liver transplantation
MC: Milan criteria
mRECIST: Modified-response evaluation criteria in solid tumors
NLR: Neutrophil-to-lymphocyte ratio
OR: Odds ratio
PIVKA-II: Protein induced by vitamin K absence-II
PLR: Platelet-to-lymphocyte ratio
PS: Patient survival
UCSF: University of California, San Francisco
